# Is the Effect of Environmental Attitudes on Behavior Driven Solely by Unobserved Heterogeneity?

**DOI:** 10.1007/s11577-022-00855-2

**Published:** 2022-10-07

**Authors:** Henrik Kenneth Andersen, Jochen Mayerl

**Affiliations:** Institute of Sociology, Chemnitz University of Technology, Thüringer Weg 9, 09126 Chemnitz, Germany

**Keywords:** Attitudes and behavior, Willingness to pay/sacrifice, Panel analysis, Fixed effects, Structural equation modeling, Einstellungen und Verhalten, Opferbereitschaft, Panelanalyse, Fixed Effects, Strukturgleichungsmodelle

## Abstract

A large body of research exists investigating the link between environmental attitudes and behavior. Many empirical studies have found modest positive effects, suggesting that attitudes toward the environment might indeed influence environmental behavior. However, most of the previous empirical work is cross-sectional and correlational in nature. This means that the issue of the causal effect of environmental attitudes on behavior is far from settled, and that the relationships observed in the past may be due to unobserved confounders. In a panel study using six waves of the GESIS Panel Survey, we examine the individual-level effect of changes in one’s attitudes on changes in different forms of environmental behavior. We use fixed effects panel regression within the structural equation modeling framework to control for unobserved time-invariant confounders, while also tackling other methodological challenges. We find that environmental attitudes have no effect on behavior after controlling for unobserved confounders. However, there is a robust effect of attitudes on willingness to sacrifice. This suggests that creating more positive attitudes might make individuals more willing to accept sacrifices for environmental protection.

## Abstract (de)

Es gibt zahlreiche Forschungsarbeiten, die sich mit dem Zusammenhang zwischen Umwelteinstellungen und -verhalten befassen. Viele empirische Studien haben meist schwach bis mäßig positive Effekte festgestellt, was darauf hindeutet, dass die Einstellung gegenüber der Umwelt tatsächlich das Umweltverhalten beeinflusst. Allerdings handelt es sich bei den meisten der bisherigen empirischen Arbeiten um Querschnitts- und Korrelationsstudien. Das bedeutet, dass die Frage nach dem kausalen Effekt von Umwelteinstellungen auf das Verhalten noch lange nicht geklärt ist und dass die in der Vergangenheit beobachteten Zusammenhänge möglicherweise auf unbeobachtete Störfaktoren zurückzuführen sind. In einer Panelstudie, die sechs Wellen der GESIS-Panel Studie verwendet, untersuchen wir die individuellen Auswirkungen von Veränderungen der eigenen Einstellungen auf Veränderungen in verschiedenen Formen des Umweltverhaltens. Wir verwenden eine Panelregression mit Fixed Effects im Rahmen der Strukturgleichungsmodellierung, um unbeobachtete zeitinvariante Störfaktoren zu kontrollieren und gleichzeitig andere methodische Herausforderungen zu bewältigen. Wir stellen fest, dass Umwelteinstellungen keinen Einfluss auf das Verhalten haben, nachdem wir für unbeobachtete Störfaktoren kontrolliert haben. Es gibt jedoch einen robusten Effekt von Einstellungen auf die Opferbereitschaft. Dies deutet darauf hin, dass eine positivere Einstellung dazu führen kann, dass die Menschen eher bereit sind, Opfer für den Umweltschutz zu bringen.

## Introduction

Fighting climate change requires not only political decisions, economic incentives, and technological progress but also, it is often argued, individual action (Engel and Pötschke 1998; Evans et al. 2007; Prati et al. 2015; Vicente et al. 2021). Individual action can mean modifying one’s behavior to, say, use the car less, buy regional and seasonal products, or eat less meat (Barr 2007). On the other hand, individuals must also be prepared to demand action by their governments and be willing to make sacrifices, potentially in the form of higher prices and taxes, in the fight against climate change.

Blake (2001) refers to these as “individual behavior” and “support for collective action” respectively, whereas Vicente et al. (2021) use the terms “private sphere behaviors” and “moderate activist behaviors in the public sphere.”^1^ Here, we will use the terms “environmental behavior” to refer to individual actions, and “willingness to sacrifice” to refer to passive support or an acceptance of collective action to preserve the environment. We will argue later that willingness to sacrifice, as it is measured here, should more appropriately be conceptualized as a type of behavioral intention.

With both of these, attitudes are often looked at as antecedents. It is plausible that the more positive one’s attitude toward the environment, the more environmentally friendly one will behave (Barr 2007; Best and Mayerl 2013; Langenbach et al. 2020) and the more willing one will be to accept sacrifices to one’s quality of life (Liebe et al. 2011; Mayerl and Best 2019; Meyerhoff 2006). If it can be convincingly established that changing environmental attitudes to be more positive does have an influence on environmentally friendly behavior, then policies should be implemented to foster such attitudes in the fight against climate change.

In a panel design, we look at the effect of environmental attitudes on two forms of individual environmental behavior: consumption and mobility behavior, as well as willingness to pay/make sacrifices. We conduct a “severe” test of whether attitudes toward the environment affect environmental behavior by applying fixed effects panel regression and examining strictly within-person processes. If we can establish a within-person link between environmental attitudes and behavior, then the policy recommendation would be to promote more environmentally friendly attitudes, by investing in information campaigns to break down anthropocentric views, for example. If there is no within-person effect, then such measures would be fruitless; changing attitudes without changing the underlying causes of both attitudes and behavior will have no effect on individual action toward the environment.

We draw data on attitudes and behavior/willingness to sacrifice in Germany from six waves (2014–2019) of the GESIS Panel study (GESIS 2020) and estimate separate statistical models for each behavior in a panel analysis using the structural equation modeling (SEM) framework. This allows us to look at individual-level changes, thereby hopefully bringing us closer to identifying the causal effect of attitudes on behavior in the context of the environment.

We proceed as follows. First, in Sect. 2, we discuss the motivation for examining environmental attitudes as a way of explaining behavioral decisions and actual behavior. We argue from a wide rational choice perspective that attitudes and constraints should have additive effects on behavioral choices, and that holding the constraints facing each individual constant is one of the keys to being able to look at the causal effect of attitudes. Then, in Sect. 3, we discuss our strategies for dealing with several other methodological challenges. In Sect. 4, we outline our dataset and describe our modeling strategy in detail. In Sect. 5, we discuss the findings of the panel SEMs. Finally, in Sect. 6, we summarize our findings and discuss implications for future work.

## Analytical Background

This article looks to further investigate the question of how to influence pro-environmental behavior. We focus on attitudes because they are often seen, besides the costs and constraints (more on that below), as one of the main predictors of environmental behavior (Best and Kneip 2011; Best and Mayerl 2013; Eagly and Chaiken 1998; Rokeach 1968). If we can firmly establish that environmental attitudes have a causal impact on environmental behavior—in the sense that when an individual develops more environmentally friendly attitudes (in isolation of other factors), that person also begins to behave in a more environmentally friendly way—then policy should focus on information campaigns, perhaps at early ages, aimed at breaking down anthropocentric views and beliefs in order to foster more positive environmental attitudes. If, on the other hand, the relationship between environmental attitudes and behavior is shown to be spurious, i.e., both caused by other unobserved variables, then the policy relevance of attempting to change individuals’ attitudes is essentially naught.

And, in fact, although the relationship between environmental attitudes and behavior has been examined in numerous studies in the past, the policy relevance of attempting to change attitudes to promote behavior is hardly settled. On the one hand, many have found links between environmental attitudes and behavior in the past. To name just a few examples, Blake (2001), Mobley et al. (2010), Schwab et al. (2014), Brick and Lewis (2014), Langenbach et al. (2020), Kesenheimer and Greitemeyer (2021) all found positive effects of environmental attitudes/concern on environmental behavior in empirical analyses. Other studies have looked specifically at the effect of environmental attitudes on willingness to sacrifice for the environment (Dienes 2015; Harring 2013; Khachatryan et al. 2014; Kyselá 2016; Liebe et al. 2011; Marbuah 2016; Mayerl and Best 2019; Meyer and Liebe 2010). Mayerl and Best (2019), for example, found positive correlations between environmental attitudes and willingness to sacrifice in 29 of the 30 countries investigated.

On the other hand, it is often observed that the link between environmental attitudes and behavior is often weaker than we might expect (Diekmann and Preisendörfer 1998). Engel and Pötschke (1998), for example, give an overview of a number of studies from the 1980s and 1990s, where associations between environmental attitudes and behavior were generally rather low, with most studies finding between 7 and 30% explained variance in behavior. Best and Mayerl (2013) note that the environmental attitude–behavior link is rather weak in most studies, as well. In their later study, the correlation between attitudes and willingness to sacrifice was quite weak in several countries, including Mexico, the Philippines, Russia, Slovenia, and Turkey (Mayerl and Best 2019).

Furthermore, the majority of research investigating the relationship between environmental attitudes and behavior can be described as cross-sectional and correlational in nature (Blankenberg and Alhusen 2018).^2^ This includes the examples mentioned above. The issue with this is that the assumptions needed to identify a causal effect with correlational (i.e., non-experimental) data are often unrealistic. In order to identify a causal effect, we need to establish:

1. A correlation between environmental attitudes and behavior,
2. Ensure temporal precedence (i.e., that the cause comes before the effect and the direction of causality is clearly from *X* to *Y*, so to speak),
3. Isolation of the independent variable from all other factors influencing the dependent variable (Bollen 1989).

In a cross-sectional setting, we may observe some correlation, but for one, the temporal precedence is impossible to establish. This means that we cannot be sure that *X* leads to *Y* or whether it is the other way around. More importantly, however, it is exceedingly difficult to establish isolation of the independent variable.

We tackle these and related issues by applying panel regression analysis within the SEM framework. We apply a type of panel fixed effects (FE) model to control for all sources of time-invariant unobserved heterogeneity, ruling out a vast number of potential confounders. We use multiple indicator measurement models to establish the validity of our measures of environmental attitudes and behavior/willingness to sacrifice and to avoid attenuation bias due to measurement error. Furthermore, we control for past behavior—essentially habits—as a second source of unobserved heterogeneity (i.e., some time-varying confounders) and look at the lagged effects of attitudes on behavior to establish temporal precedence. This allows us to control for a wide range of time-varying confounders, even if they remain unobserved, as we will discuss below.

In the next section, we lay out the theoretical argument for focusing on attitudes as the main explanatory variable and then move on to addressing the methodological challenges associated with establishing a causal effect with survey panel data.

### Theoretical Explanation

Best and Kneip (2011) suggest that a thorough investigation of environmental behavior should consider both cost-benefit aspects as well as attitudes. From a rational choice perspective, actors behave based on their preferences within the constraints that restrict the alternatives from which they can choose. Constraints refer to the costs associated with a given action, i.e., the effort associated with bringing recyclables or glass to a drop-off point, or taking the bike instead of the car (Best and Kneip 2019). Preferences usually pertain to those “hard” factors like time and money.^3^ In the context of environmental behavior, however, it is not always easy to identify hard preferences for environmentally friendly action; no one offers to pay if you recycle instead of toss everything into the trash, it actually requires more physical effort to ride one’s bike to work than to use the car, and ecologically friendly products are almost always more expensive than conventional products. This means that in most cases, behaving in an environmentally friendly way is costlier than behaving in an environmentally unfriendly way, so a narrow interpretation of rational choice would suggest that it might rarely be rational to do so. Therefore, in the context of environmental behavior, a “wide” interpretation of rational choice considers behavior based on attitudes as having “intrinsic utility” (Best and Kneip 2011; Mayerl 2009; Opp 1999). In other words, pro-environmental actors can choose to behave based on attitudes to fulfill their preference of behaving in an attitude-conform manner, thereby avoiding cognitive dissonance (Festinger 1957), for example.

This means there are at least two categories of predictors of environmental behavior: those constraints (costs) that each individual faces when deciding how to behave, and attitudes as preferences. We can assume that there is a distribution of constraints as well as attitudes in the population (Best and Kneip 2019). That is, in terms of constraints, some live close to their place of work, so riding a bike is less costly to some than it is for others. Some individuals earn enough money to be able to more easily afford ecologically friendly products than others, etc. In terms of preferences, some have strong positive attitudes toward the environment, some may be ambivalent, and some may see economic and environmental goals as being at odds with each other and even have a strong preference for the economic at the cost of the environmental.

The rational choice perspective suggests an additive effect of these two categories of predictors (Best and Kneip 2011): those with favorable conditions (living close to work, or close to an ecological supermarket, or with more monetary resources) can pursue environmentally friendly behavior more “easily” than others. And attitudes are assumed to affect behavior over and above these constraints: for two given individuals with otherwise identical constraints, the one with the more positive environmental attitudes should behave in a more environmentally friendly way. This suggests two approaches to modifying behavior: based on the argument concerning constraints, more environmentally friendly behavior can be achieved by reducing costs, e.g., switching to curbside recycling pickup as opposed to asking individuals to physically take their recyclables to drop-off points. On the other hand, promoting environmentally friendly attitudes should increase environmentally friendly behavior by making environmentally unfriendly behavior less preferable, i.e., owing to cognitive dissonance, as attitudes become more positive, environmentally unfriendly behavior becomes more uncomfortable.

In this study, we attempt to *isolate the effect of preferences from constraints*, focusing on the former while holding the latter constant statistically. And it bears emphasizing: when we examine the effect of attitudes on behavior, what we are essentially looking at is whether a *change in preferences *has an effect on a *change in behavioral choices*, which differs from the usual procedure of looking at *changes in resources/constraints* as a source of behavioral change. The problem in empirically assessing the question of whether a change in preferences leads to a change in behavior is that it can quickly dissolve into a circular argument, i.e., it is tempting but ultimately pointless to attempt to infer a change in attitudes from a change in behavior (Opp 2020; Diekmann and Voss 2004). Therefore, we need to be rigorous in our analytical and statistical approaches, to ensure that we are looking at the isolated effect of (an individual-level change in) preferences or rather attitudes on (an individual-level change in) behavior. The next section will outline our design, which is focused on the validity of our measures, as well as accounting for both time-invariant and time-varying potential confounders.

## Design

In this section, we touch on a number of methodological issues and discuss our strategies for addressing them. This involves two broad categories. First, in order to avoid circular arguments, we must establish the valid and reliable measurement of both of the constructs of interest, so that we can say we have measured both attitudes and behavior/willingness to sacrifice and that these are separate constructs whose meanings remain stable over time. Second, we must establish an individual-level model of behavioral change. We want to identify the effect of a change in one’s individual attitudes on their own behavior/willingness to sacrifice, while controlling for a range of potential confounding through unobserved differences in resources.

We will begin by discussing the measurements in order to address the issue of circular arguments. Following that, we will discuss our strategies for isolating the effect of attitudes on behavior.

### Measurements

The independent variable in our study is a measure of environmental attitudes, drawn from the New Environmental Paradigm (NEP) scale (Dunlap et al. 2000). This has been used widely throughout the literature to operationalize generalized environmental attitudes (Amburgey and Thoman 2012; Best and Mayerl 2013; Mayerl and Andersen 2018; Mayerl and Best 2019).

The mobility behavior items were taken from the so-called “Mobilität in Deutschland 2002 Personen- und Wegefragebogen” (English: “Mobility in Germany 2002 People and Routes Survey”); the consumption behavior items were constructed for the GESIS Panel study. Both mobility and consumption behavior are operationalized in terms of retrospective behavioral reports. Respondents were asked to report how often they purchased regional or organic foods in the recent past (the last week before the survey), as well as how often they tend to use different modes of transportation.

Willingness to sacrifice for the environment can be thought of as “the extent to which individuals’ decisions will take into account the well-being of the environment, even at the expense of immediate self-interest, effort, or costs” (Davis et al. 2011). Although the word “decision” implies behavior, willingness to sacrifice is most often seen as a type of *behavioral intention* (Klösch et al. 2021; Knez et al. 2020; Mayerl and Best 2019), which, following the Theory of Planned Behavior (Fishbein and Ajzen 1972), is seen to mediate the effect of attitudes on behavior. Thus, willingness to sacrifice is a sort of precursor to behavior and it is something distinct from environmental attitudes. As Mayerl and Best state, “environmental attitudes should be conceptualized as the predictor of environmental intentions” (Mayerl and Best 2019, p. 30).

It is also important to note that willingness to sacrifice captures something distinct from individual behavioral choices, like those having to do with mobility and consumption. Indeed, the items used to measure willingness to sacrifice in the GESIS Panel and were taken from the International Social Survey Programme (ISSP) 2010. These items highlight a *passive acceptance* of collective decisions. They ask whether the respondent is willing to pay higher taxes and prices, and accept a sacrifice to their lifestyle to protect the environment. This is different than asking respondents about whether they intend to make sacrifices to their lifestyle for the environment, which would suggest individual behavioral decision making.

Conceptualizing willingness to sacrifice as a type of behavioral intention may seem unsatisfying, at first: just because someone states that they intend to behave in a certain way, does not guarantee that they will follow through. But again, it is important to note that willingness to sacrifice captures a passive acceptance of collective decisions. One cannot set his/her own tax rate irrespective of income, and the item concerning higher prices makes no reference to active consumer decisions. It simply asks whether the respondent is willing to pay higher prices “in order to protect the environment” in general. To summarize, we consider willingness to sacrifice as a precursor to behavior. It tells us how individuals intend to act with regard to collective decisions (e.g., accept them, or protest them). They are distinct from, and indeed the result of, environmental attitudes.

In a first step, we examined the psychometric properties of the measurements of each of the constructs using confirmatory factor analysis (CFA) in SEM.^4^ In an iterative fashion, we ran several CFAs for each construct (environmental attitudes, consumption and mobility behavior, and willingness to sacrifice) paring down the indicators in some cases until we found satisfactory psychometric properties while retaining the strong longitudinal invariance assumptions. The final measurement models consist in the operationalizations shown in Tab. 1. In each case, the measurement models under strong measurement invariance (factor loadings and item thresholds held constant over time, see for example Liu et al. (2016)) are largely satisfactory, with comparative fit indices (CFI) larger than 0.95, and root mean squared error of approximation (RMSEA) and standardized root mean squared residual (SRMS) statistics usually within the range 0.1–0.7.^5^ The Chi-squared statistic, on the other hand, is substantial and significant in each case, signaling non-ignorable misfit. However, we are satisfied in most respects with the psychometric properties of the scales, and our overall goal is not to test an all-encompassing theory of behavior, but rather to focus on the rigorous estimation of the main effect of interest, concerning the effect of attitudes on behavior/willingness to sacrifice.

**Table 1:** Construct list and operationalization

Latent construct	Label	Wording	Scale
Consumption behavior	Shpbiomrkt	Did you buy any organic food during the past week, that is, food of controlled organic cultivation?	1: No, none, 2: Yes, sometimes, 3: Yes, (almost) exclusively
	Shprgnlprdct	Did you buy any fruits and vegetables from regional producers during the past week, that is, fruit and vegetables that were cultivated in your region?	1: No, none, 2: Yes, sometimes, 3: Yes, (almost) exclusively
Mobility behavior	Usgsbusrgn^a^	Please specify how often you normally use the following means of transportation: regional bus or train	1: (Almost) daily, 2: On 1 to 3 days per week, 3: On 1 to 3 days per month, 4: Rarer, 5: (Almost) never
	Usgsbuslngrng^a^	Please specify how often you normally use the following means of transportation: train for longer distances	1: (Almost) daily, 2: On 1 to 3 days per week, 3: On 1 to 3 days per month, 4: Rarer, 5: (Almost) never
	Usgscar	Please specify how often you normally use the following means of transportation: car	1: (Almost) daily, 2: On 1 to 3 days per week, 3: On 1 to 3 days per month, 4: Rarer, 5: (Almost) never
	Usgsbike^a^	Please specify how often you normally use the following means of transportation: bike	1: (Almost) daily, 2: On 1 to 3 days per week, 3: On 1 to 3 days per month, 4: Rarer, 5: (Almost) never
Willingness to sacrifice	Wtshrprcs^b^	How willing would you be to pay much higher prices in order to protect the environment?	1: Very willing, …, 5: Very unwilling
	Wtshrtxs^b^	How willing would you be to pay much higher taxes in order to protect the environment?	1: Very willing, …, 5: Very unwilling
	Wtslfstl^b^	And how willing would you be to accept cuts in your standard of living in order to protect the environment?	1: Very willing, …, 5: Very unwilling
Environmental attitudes	Nepadptenvrnmt^c^	Humans have the right to modify the natural environment to suit their needs	1: Fully agree, …, 5: Fully disagree
	Nepblncntr^c^	The balance of nature is strong enough to cope with the impacts of modern industrial nations	1: Fully agree, …, 5: Fully disagree
	Nepenvcrsisexgrt^c^	The so-called “ecological crisis” facing humankind has been greatly exaggerated	1: Fully agree, …, 5: Fully disagree
	Nephmrlntr^c^	Humans were meant to rule over the rest of nature	1: Fully agree, …, 5: Fully disagree

^a^Recoded so that larger values indicate more environmentally friendly transportation behavior

^b^Recoded so that larger values indicate more willingness to sacrifice

^c^Recoded so that larger values indicate more environmentally friendly attitudes

The proportion of within-person variance for each construct usually varied between 10 and 20% of the overall variance (at the latent variable level). Willingness to sacrifice displayed the most within-person variance (around 20–30% depending on the particular point in time), whereas consumption behavior displayed almost no within-person variance at the first two points in time (2015, 2016), with more (around 13–25%) at the subsequent points in time. This means that there is some variation within individuals over time in terms of attitudes and behavior, and that we can move forward with the longitudinal models that try to explain within-person variation of behavior with that of attitudes.

Furthermore, descriptive statistics of the observed items (averaged over all points in time) are shown in Tab. 2. These are based on the pooled long-format data, with repeated measures per individual stacked on top of one another (*n* x t stands for person-years). We can see from the fact that the number of valid responses differ per item that the panel is unbalanced.

**Table 2:** Descriptive statistics, pooled data set

Latent construct	Observed variable	*n* x t	Missing	Mean	Median	SD
Consumption behavior	Shpbiomrkt	24,523	728	1.85	2.00	0.55
	Shprgnlprdct	24,063	1188	2.10	2.00	0.55
Mobility behavior	Usgsbusrgn	23,895	1356	3.81	4.00	1.34
	Usgsbuslngrng	23,621	1630	4.36	4.00	0.78
	Usgscar	24,485	766	1.77	1.00	1.16
	Usgsbike	23,605	1646	2.97	3.00	1.51
Willingness to sacrifice	Wtshrprcs	25,114	137	3.60	4.00	0.88
	Wtshrtxs	24,740	511	2.99	3.00	1.03
	Wtslfstl	25,053	198	3.62	4.00	0.83
Environmental attitudes	Adptenvrnmt	24,964	287	2.58	2.00	1.00
	Nepblncntr	25,028	223	2.08	2.00	0.84
	Nepenvcrsisexgrt	24,928	323	2.27	2.00	0.95
	Nephmrlntr	24,939	312	2.12	2.00	0.94

Descriptive statistics based on original scales, see Tab. 1

*SD* standard deviation

### Unobserved Time-Invariant Confounders

As mentioned in the previous section, both constraints and attitudes should play a role in influencing behavior. However, if constraints and attitudes both influence behavior, and if the constraints and attitudes are correlated, then constraints are confounders of the effect of attitudes on behavior and must be dealt with. Obviously, there are a great number of potential behavioral constraints facing each individual. If we are interested in environmentally friendly mobility behavior, for example, then where someone lives, their age and fitness, family situation, and upbringing, are all things that effectively restrict the pool of behavioral alternatives. Moreover, many of these aforementioned examples of constraints are likely confounders. For example, it is easy to imagine that those who live in more suburban or rural areas have a longer distance to travel to work, on average, making the car preferable or even indispensable. It is also plausible that those who live in these regions tend to be more conservative and thus less concerned about the environment as well (Dunlap 1975). Thus, in this example, region is very likely a confounder of the effect of environmental attitudes on transportation behavior. The difficulty with addressing constraints is the sheer number of potential variables. However, we can look to analytical methods that can make it possible to account for these unobserved confounders.

Dealing with time-invariant unobserved confounders is the strength of fixed effects regression. Fixed effects regression refers to a number of strategies for controlling for unobserved stable confounders. Essentially, with panel (or hierarchical) data, we can decompose the usual error term into a part that is constant over time and a part that changes over time (Andersen 2022; Bollen and Brand 2010). The part that is constant over time is referred to as time-invariant unobserved heterogeneity, the combined effect of all the factors influencing the dependent variable that themselves do not change over time: year of birth, region, upbringing, values, etc. Even things that can potentially change but are relatively stable over the course of observation can be included in the time-invariant unobserved heterogeneity, such as health, motivation, family, or job situation.^6^

We apply fixed-effects regression within the SEM framework to control for these sources of time-invariant heterogeneity, even though we do not have data on them. This works by specifying a latent variable that influences the dependent variable at all points in time. This represents the time-invariant portion of the error term. We allow this latent variable to covary with the main predictor of interest, environmental attitudes, to eliminate potential confounding from these factors.

### Unobserved Time-Varying Confounders

Fixed-effects regression allows us to deal with time-invariant confounders. However, time-varying confounders can bias the effects of interest as well. We could attempt to identify and control for all potential time-varying confounders. This comes with at least two problems. First, we must be capable of identifying all the possible time-varying confounders of the effect of environmental attitudes on behavior. We can certainly identify some time-varying confounders based on domain knowledge and previous research, but we will surely not be able to catch all of them. Second, as we are analyzing secondary data, we are forced to work within the limits of the data available. There will likely be some time-varying confounders that we simply do not have measures on.

**Fig. 1:**
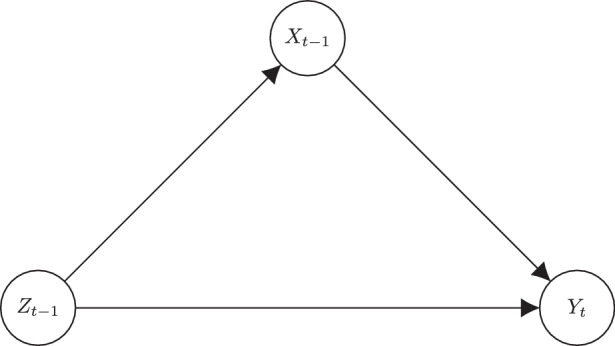
Directed graph, confounding by unobserved variable *z*_*t*−1_

So, we look to deal with time-varying confounders in another way. Consider the directed graph/path diagram shown in Fig. 1, where the lagged independent variable, environmental attitudes, represented by *x*_*t*−1_, affects the current measure of the dependent variable, environmental behavior, represented by *y*_*t*_. Let us assume that there is some other variable, *z*_*t*−1_, that influences both *x*_*t*−1_ and *y*_*t*_ and say it is unobserved. The fact that *z*_*t*−1_ is an unobserved confounder means that the effect $x_{t-1}\rightarrow y_{t}$ will be biased.

**Fig. 2:**
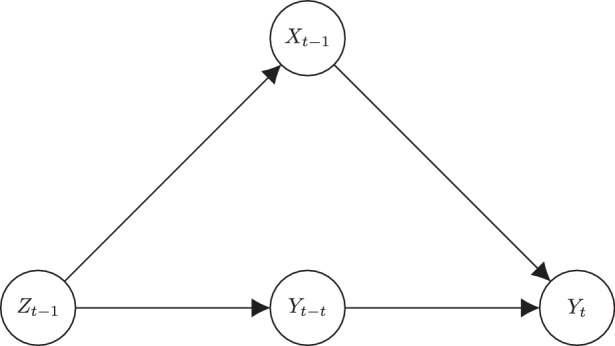
Directed graph, closing the back door path with lagged dependent variable *y*_*t*−1_

If we can assume, however, that the unobserved variable *z*_*t*−1_ affects the lagged value of *y* as well (see Fig. 2), and that after controlling for *y*_*t*−1_, the unobserved confounder has no further direct effect on *y*_*t*_, then the inclusion of *y*_*t*−1_ can be said to “close the back door path” $y_{t}\leftarrow z_{t-1}\rightarrow x_{t-1}$, to use the terminology of Pearl (2000). Thus, by including the individual’s previous behavior in the prediction of the current behavior—essentially by taking habits into account—we avoid bias from certain time-varying confounders (Kühnel and Mays 2019).

So, including the lagged dependent variable can potentially eliminate confounding by time-varying unobserved variables. This depends on the assumption that the lagged dependent variable fully mediates the effect of *z*_*t*−1_ (besides the other indirect path over *x*_*t*−1_) but it is not immediately clear why some time-varying confounders should have a direct effect on the outcome over and above the lagged version.

**Fig. 3:**
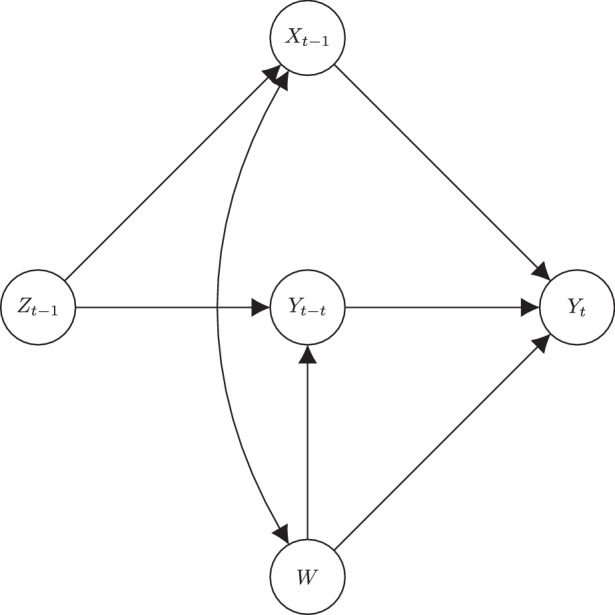
Directed graph, confounding due to time-invariant unobserved heterogeneity *w*

Although the inclusion of the lagged dependent variable has the desirable effect of blocking back-door paths of time-varying confounders, they are not uncontroversial. One of the most convincing arguments against the use of lagged dependent variables comes from Morgan and Winship (2015, p. 111, Fig. 4.3). There, they describe how the inclusion of the lagged dependent variable can be a collider and open up a second, unintended back-door path. This is recreated in Fig. 3 with the variable labelled *w* and it represents the *time-invariant unobserved heterogeneity*. Indeed, if there is some unobserved variable that affects the outcome at all points in time, then including the lagged dependent variable will bias the effect of interest, $x_{t-1}\rightarrow y_{t}$. However, Morgan and Winship (2015) describe a situation in which *w* (there, it is labelled *U*) is unobserved. It is unobserved in our models, too, except we are explicitly accounting for this source of unobserved time-invariant unobserved heterogeneity in the form of a latent variable. The SEM approach to panel analysis with lagged dependent variables is thus robust to this potential source of confounding, and the effect of interest can be estimated correctly (given that the rest of the causal diagram holds).

To summarize, we want to control for both sources of confounding, *z*_*t*−1_ and *w*, by including the lagged dependent variable in the equation for the current one, while also explicitly accounting for the time-invariant unobserved heterogeneity and the possibility that it may correlate with the lagged independent variable. In doing so, we can block both back door paths and the effect of interest, $x_{t-1}\rightarrow y_{t}{,}$ can be identified under less restrictive assumptions, i.e., that only the contemporaneous error term (which encompasses all the unobserved factors influencing *y*_*t*_ at only that specific point in time) does not correlate with the lagged independent variable. Again, the inclusion of the lagged dependent variable should rule out a large number of potential time-varying confounders.^7^

### Temporal Lags

Something Leszczensky and Wolbring refer to as an “open question” (Leszczensky and Wolbring 2022, p. 839) is the correct specification of temporal lags in panel models. Dormann and Griffin echo this sentiment, stating that “researchers have little practical guidance for choosing appropriate time lags in longitudinal studies” (Dormann and Griffin 2015, p. 489). Especially in the cross-lagged panel model tradition, the effect of the lagged independent variable is usually investigated. This makes sense insofar as the cause should logically come before the outcome. The issue, however, has to do with what constitutes a single “lag.” This depends entirely on the frequency with which the data are collected. For some studies, a lag could equal a single day. In others, such as this investigation, a lag could equal a whole year.

In terms of the relationship between attitudes and behavior, it is difficult to pinpoint how long it takes for behavioral changes to unfold after attitudinal changes occur. Likely, changes in behavior do not occur contemporaneously along with changes in attitudes. However, it is questionable as to whether it takes a whole year for the effect to unfold. Though, as Leszczensky and Wolbring (2022) note, if the effect is expected to unfold in a matter of days or even weeks, it may still be more appropriate to look at contemporaneous effects if the alternative (e.g., a lag comprising a year) is even less plausible. The opposite should be true, as well: if the effect is expected to unfold many months later, it may be better to look at the 1‑year lagged effect than the contemporaneous one. It is also plausible that both past and current realizations of the independent variable might affect the outcome.

Based on our intuition that behavioral change does not follow attitude change immediately, as well as the standardized autoregressive effects in each of the models (see below), we assume following Dormann and Griffin (2015), that looking at the 1‑year lagged relationship of attitudes on behavior is more appropriate than the contemporaneous effect.

## Data and Methods

We use the GESIS Panel study (GESIS 2020) a probability-based mixed mode (self-administered questionnaire, online and offline) access panel of the German-speaking population aged 18 to 70 (as of recruitment) with permanent residency in Germany. The data we use to operationalize environmental attitudes and behavior/willingness to sacrifice are drawn from six waves^8^, which are collected from the same individuals yearly from 2014 to 2019.^9^ Refreshment samples to compensate for sample panel attrition were conducted in 2016 and 2018. Attrition rates are reported in the GESIS Panel Wave Report (Wave gc)^10^ for the initial cohort (2013) and the first refreshment cohort (2016). These are calculated as the remaining sample size of the cohort divided by the original size, and are 42.4% for the 2013 cohort and 30.6% for the 2016 cohort.^11^

The measurements have been discussed previously in the Measurements section, so we can move on to the modeling strategy.

### Modeling Strategy

The basic model setup can be described as

$$\text{behavior}_{it}=\gamma \text{attitudes}_{it-1}+\alpha _{i}+\epsilon _{it}$$

where behavior_*it*_ is the behavior/behavioral intention in question (consumption, mobility, willingness to sacrifice) for individual $i=1{,}\ldots {,}n$ at time $t=2014{,}\ldots {,}2019$. The lagged independent variable attitudes_*it*−1_ is the same in all models and connected to behavior by the coefficient *γ*. We have no a priori reason to believe that effects should change for all individuals over time; thus, we constrain them to be equal in all time periods (equivalent to running a regression on the long-format stacked data). Both variables of interest, attitudes and behavior, are modeled at the latent-variable level to account for measurement error. Refer to Appendix: Results of CFAs for details on the measurement models.

The variable *α*_*i*_ is a latent variable that affects behavior at all points in time. This accounts for the time-invariant unobserved heterogeneity; those unobserved factors that cause stable differences in individuals’ behavior over time. Finally, *ϵ*_*it*_ is the normal time-varying error term. For the dynamic models, we add the lagged dependent variable to account for time-varying confounders:

$$\text{behavior}_{it}=\gamma \text{attitudes}_{it-1}+\beta \text{behaviour}_{it-1}+\alpha _{i}+\epsilon _{it}.$$

**Fig. 4:**
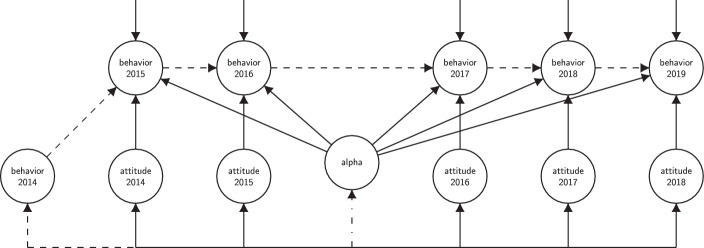
Full dynamic fixed-effects model. (Baseline model is RE. FE: Correlation between attitudes and alpha (*dash-dotted*). DFE: Autoregressive effect (*dashed*). “alpha” is a latent variable representing time-invariant unobserved heterogeneity. Measurement models not shown due to space constraints, see Appendix for details)

We begin with the Random Effects (RE) model, which respects the fact that there will likely be unobserved factors causing stable differences between individuals over time but assumes that these stable factors do not correlate with attitudes. This is a highly improbable assumption, but it allows us to assess the extent to which a more correlational approach may have led us astray. The fixed effects (FE) model loosens this assumption by allowing the unobserved stable factors and attitudes to be correlated. This accounts for confounding by time-invariant factors. Further, the Dynamic Fixed Effects (DFE) model includes the previous measure of behavior in the equation for current behavior. As discussed above, this should account for further time-varying confounders.^12^ Figure 4 shows our modeling strategy within the SEM framework.

## Results

We will discuss the following models by looking at each of the specifications individually, starting with the RE model. Note that the RE model is nested within the FE one where the correlations between the covariates and the latent individual effects are set to zero. The FE model is not nested within the DFE model; however, because behavior in 2014, which is used to predict behavior in 2015, does not appear in the FE model. We will return to this point in the willingness to sacrifice models, because it will become particularly relevant there. This means that we can test the assumptions of the specifications empirically by comparing the RE and FE models using likelihood ratio (sometimes: Chi-squared difference) tests. This will help to guide our decisions on which models to focus our substantive interpretations on.

### Consumption Behavior

The RE model assumes that there are unobserved time-invariant factors influencing consumption behavior (shopping at organic food stores, buying regional and seasonal products), but that these factors do not correlate with attitudes. If this assumption does not hold—we assume that it will not hold as many unobserved stable characteristics will correlate with attitudes—then the effect of attitudes on behavior will be biased. In the RE model, we get a highly significant positive estimated effect of 0.512*** (SE: 0.037, 95% CI: [0.439, 0.584]), with standardized effects ranging from 0.353 to 0.395 depending on the particular point in time, see Tab. 3.^13^ The more positive the attitudes, the more environmentally friendly the reported behavior is. This is in line with many of the cross-sectional correlational findings in the past.

In the FE model, we assume that the unobserved stable characteristics do indeed correlate with attitudes. The FE model thus partials out this source of confounding and gives us a more accurate view of the within-unit effect, which can be interpreted as the effect of a person’s own attitudes growing more positive (compared with their “usual levels”) on their own behavior relative to their “usual” behavior. In terms of consumption behavior, we see in the FE model that the effect is much lower and no longer statistically significant at 0.064 (SE: 0.067, 95% CI: [−0.066, 0.194]) with standardized effects ranging from just 0.044 to 0.050. A person’s environmental attitudes becoming more positive does not seem to have a significant effect on their own consumption behavior. The results of the likelihood ratio test indicate that the FE model fits significantly better than the RE one, with a Chi-squared difference of 16.972** (5 degrees of freedom difference).

The DFE model adds to the FE model an autoregressive effect of past behavior on current behavior. We can look at these effects in terms of habits, or more abstractly as a way of controlling for time-varying confounders. In this model, the autoregressive effect is positive and significant at 0.408** (SE: 0.132, 95% CI: [0.149, 0.668]), with standardized effects of 0.377 to 0.420. If a person’s behavior became more environmentally friendly during the last period, it tends to positively impact their behavior in the subsequent period as well. Barr (2007) referred to this as the “behavioral snowball effect” and it is supported in the consumption behavior model. The main effect of interest, however, is not significantly different than zero. It is estimated at −0.003 (SE: 0.060, 95% CI: [−0.120, 0.114]) with standardized effects of −0.002 during all time periods.

### Mobility Behavior

Moving on to the mobility behavior models, we see that the effects largely mirror those seen in the consumption models, see Tab. 4. In the RE specification, the effect of attitudes on mobility behavior is significant and positive at 0.175*** (SE: 0.040, 95% CI: [0.098, 0.253]), with standardized effects ranging from 0.137 to 0.148 depending on the particular point in time.

**Table 3:** Regression results, consumption behavior

		RE			FE			DFE		
		Estimate	SE	*P* (> |z|)	Estimate	SE	*P* (> |z|)	Estimate	SE	*P* (> |z|)
IV	DV									
EA (t‑1)	Cons (t)	0.512***	0.037	0.000	0.064	0.067	0.335	−0.003	0.060	0.962
Cons (t‑1)	–	–	–	–	–	–	–	0.408**	0.132	0.002
*n*		1775			1775			1720		
Chisq (df)		1579.303*** (429)			1580.106*** (424)			1591.171*** (471)		
CFI		0.978			0.978			0.979		
RMSEA		0.039			0.039			0.037		
SRMR		0.040			0.039			0.038		

Estimator: Diagonally weighted least squares; robust standard errors and mean- and variance-adjusted test statistic

*IV* independent variable, *DV* dependent variable, *EA* environmental attitudes, *Cons* consumption behavior, *RE* Random Effects, *FE* Fixed Effects, *DFE* Dynamic Fixed Effects, *SE* Standard Error, *CFI* Comparative Fit Index, *RMSEA* Root Mean Squared Error of Approximation, *SRMR* Standardized Root Mean Square Residual

*p < 0.05, **p < 0.01, ***p < 0.001

**Table 4:** Regression results, mobility behavior

		RE			FE			DFE		
		Estimate	SE	*P* (> |z|)	Estimate	SE	*P* (> |z|)	Estimate	SE	*P* (> |z|)
IV	DV									
EA (t‑1)	Mob (t)	0.175***	0.040	0.000	−0.049	0.045	0.280	−0.031	0.054	0.569
Mob (t‑1)	–	–	–	–	–	–	–	0.908***	0.114	0.000
n		1517			1436			1436		
Chisq (df)		2433.173*** (800)			2443.581*** (795)			2902.133*** (949)		
CFI		0.985			0.985			0.984		
RMSEA		0.037			0.037			0.038		
SRMR		0.058			0.058			0.061		

Estimator: Diagonally weighted least squares; robust standard errors and mean- and variance-adjusted test statistic

*IV* independent variable, *DV* dependent variable, *EA* environmental attitudes, *Mob* mobility behavior, *RE* Random Effects, *FE* Fixed Effects, *DFE* Dynamic Fixed Effects, *SE* Standard Error, *CFI* Comparative Fit Index, *RMSEA* Root Mean Squared Error of Approximation, *SRMR* Standardized Root Mean Square Residual

*p < 0.05, **p < 0.01, ***p < 0.001

Again, however, it appears that this effect is potentially spurious, as the effect is much smaller in the FE model with a nonsignificant estimated effect of −0.049 (SE: 0.045, 95% CI: [−0.138, 0.040]), with standardized effects ranging from −0.038 to −0.042. A person whose attitudes become more positive does not seem to change their mobility behavior relative to their typical ways. Interestingly, however, the results of the likelihood ratio comparison show a nonsignificant Chi-squared difference of just 9.0113 (5 degrees of freedom difference). This actually indicates that the FE model does not fit significantly better than the RE one, and we could perhaps retain the assumption that the individual effects do not correlate with attitudes.

This remains the case in the DFE model, where the effect of attitudes remains nonsignificant with an estimate of −0.031 (SE: 0.054, 95% CI: [−0.136, 0.075]) and standardized effect of between −0.024 and −0.026, whereas the autoregressive effect is positive and highly significant at 0.908*** (SE: 0.114, 95% CI: [0.685, 1.131]). Here, it is worth emphasizing the standardized effect estimates: these range from 0.884 to 0.923, which are extremely large. This means that there is considerable dependency over time in one’s mobility behavior and we could say that it is highly habitual in nature. It seems plausible that the reason why the FE model did not fit significantly better than the RE model is that the source of the empirical regularity in mobility behavior is not due to unobserved stable characteristics but rather fully explained by habits, considering the extremely large standardized autoregressive effects. For this reason, we prefer to interpret neither the RE nor the FE model substantively, but rather the DFE.

### Willingness to Sacrifice for Environmental Protection

Finally, we move on to the willingness to pay or sacrifice models shown in Tab. 5. In the RE specification, we observe a positive and significant effect of environmental attitudes on willingness to sacrifice in the subsequent period, with an estimated effect of 0.712*** (SE: 0.030, 95% CI: [0.654, 0.771]). More positive attitudes lead to greater willingness to sacrifice. The standardized effects are quite large in the case of willingness to sacrifice as well, ranging from 0.478 to 0.487.

**Table 5:** Regression results, willingness to sacrifice

		RE			FE			DFE			DFEm		
		Estimate	SE	*P* (> |z|)	Estimate	SR	*P* (> |z|)	Estimate	SE	*P* (> |z|)	Estimate	SE	*P* (> |z|)
IV	DV												
EA (t‑1)	Wts(t)	0.712***	0.030	0.000	0.119*	0.053	0.025	−0.026	0.055	0.633	0.092*	0.040	0.021
Wts (t‑1)	–	–	–	–	–	–	–	0.307***	0.028	0.000	0.008	0.015	0.589
n		1936			1936			1898			1936		
Chisq(df)		4233.369*** (610)			4134.525*** (605)			4407.001*** (702)			4168.399*** (604)		
CFI		0.946			0.948			0.948			0.947		
RMSEA		0.055			0.055			0.053			0.055		
SRMR		0.060			0.058			0.056			0.058		

Estimator: Diagonally weighted least squares; robust standard errors and mean- and variance-adjusted test statistic

*IV* independent variable, *DV* dependent variable, *EA* environmental attitudes, *Wts* willingness to sacrifice, *RE* Random Effects, *FE* Fixed Effects, *DFE* Dynamic Fixed Effects, *DFEm* Dynamic Fixed Effects (modified to be nested in DFE), *SE* Standard Error, *CFI* Comparative Fit Index, *RMSEA* Root Mean Squared Error of Approximation, *SRMR* Standardized Root Mean Square Residual

*p < 0.05, **p < 0.01, ***p < 0.001

Uniquely, the effect of attitudes on willingness to sacrifice does not “disappear” in the FE model as it did with consumption and mobility behavior. Although it drops dramatically compared with the RE specification, the effect remains positive and significant at 0.119* (SE: 0.053, 95% CI: [0.015, 0.223]), with standardized effects ranging from 0.081 to 0.083. This suggests that when a person’s attitudes become more positive (again, relative to their “usual” attitudes), then their willingness to sacrifice might increase as well (relative to their “usual” willingness). The Chi-squared difference is significant at 16.972** (5 degrees of freedom difference), indicating that the FE specification is supported by the data more than the RE one is.

In the DFE model, the estimated effect drops slightly below zero and becomes nonsignificant at −0.026 (SE: 0.055, 95% CI: [−0.135, 0.082]), with standardized effects of −0.018 in all time periods. The autoregressive effect is again positive and significant at 0.307*** (SE: 0.028, 95% CI: [0.253, 0.361]), with standardized effects ranging from 0.298 to 0.310. A change in willingness to sacrifice at one point in time affects willingness at the next point in time as well.

Up until now, we were unable to compare the FE and DFE models because they were not nested. This is troubling because there is a discrepancy: the FE model suggests a significant positive effect of attitudes on behavior, but the DFE model, which controls for previous behavior, suggests otherwise. If we drop the initial lagged measure of behavior in 2014, the two models become nested with one degree of freedom difference (owing to the autoregressive effect, which is held constant over time) and we can use the likelihood ratio test to investigate whether the more restrictive model (FE) fits significantly worse than the DFE. If it does not, then it would indicate that we should focus our substantive interpretation on the FE model, as its assumptions can be retained.

We do so in a fourth model for willingness to sacrifice (DFEm), whose results are also reported in Tab. 5. In this model, the effect of attitudes on behavior is again significant and positive at 0.092* (SE: 0.04, 95% CI: [0.014, 0.171]). The likelihood ratio test, however, does not support the model with lagged dependent variable effects, with a nonsignificant Chi-squared difference of just 0.266. For this reason, we prefer to interpret the results of the FE model. From this, we can conclude that the weak but positive significant effect of attitudes on behavior is relatively robust in terms of willingness to sacrifice. A change in an individual’s attitudes to become more positive at one point in time leads to an increase in one’s willingness to sacrifice relative to their usual willingness.

## Discussion and Conclusion

In this article, we investigated on a within-individual-level whether a change in attitudes toward the environment can have an impact on that person’s behavior at a later point in time, or whether the association is driven by unobserved heterogeneity. We investigated three types of environmental behavior: consumption, transportation, and willingness to sacrifice. Although past literature has reported small to moderate correlations between environmental attitudes and behavior, we find that in terms of everyday behavior, these relationships may be spurious. Namely, the random effects (RE) specifications we ran do not control for unobserved time-invariant heterogeneity and thus returned upwardly biased positive effects of attitudes on behavior in all cases. In the case of consumption and mobility behavior, as soon as we switched to the fixed effects (FE) specification—essentially holding those unobserved confounders constant—we saw that the effects “disappear” and do not return in the subsequent model specification. We can conclude based on this that a change in environmental attitudes likely *does not *cause individuals to change their everyday behavior.

This finding is troubling to some extent, because it implies that attempts to simply raise awareness of problems facing the environment will not work to encourage individuals to behave in a more environmentally friendly way. But if attitudes do not change behavior, then what can be done? Going back to the arguments sketched above, then it seems logical to look at the other assumed predictors of behavior: the constraints. Indeed, in their panel analysis Best and Kneip (2011) found that reducing behavioral constraints had a larger effect on pro-environmental than attitudes.^14^ Policy makers must focus on the arguably more challenging task of actually reducing barriers to environmentally friendly behavior. However, as mentioned in the introduction, we find it reasonable to differentiate between individuals making everyday choices of their own accord and individuals passively accepting decisions brought down on them from above. In fighting climate change, both of these aspects likely play a meaningful role. And, in fact, the effect of environmental attitudes on willingness to sacrifice is relatively robust in our model specifications. Although the effects are modest in size, they are significant and positive (in the RE, FE, and dynamic fixed effects (DFEm) models). If an individual’s attitudes become more positive relative to their own usual attitudes, then their willingness to sacrifice (measured in terms of higher taxes, higher prices, sacrifices in their life) increases as well, relative to their usual willingness. This is in line with the findings in the literature noted above of small to moderate positive effects. Our results show, however, that correlational analyses likely overestimate the effect of attitudes on willingness to pay.

We therefore draw the conclusion that if fighting climate change relies on governments enforcing necessary measures (e.g., eventually banning the production of new fossil fuel-burning cars, higher taxes, and subsidies for alternative energy and mobility solutions), then it will be prudent to prime individuals for these top-down decisions by promoting positive attitudes toward the environment. This may not lead to individuals voluntarily changing their everyday behavior in a meaningful way, but it may make them more accepting of any discomfort the measures may bring.

If environmental attitudes do, in fact, make individuals more willing to accept the negative consequences of having to fight climate change, then the question remains: how do we change attitudes? There is a wide body of literature on the topic of attitude change,^15^ and many approaches to changing attitudes have been suggested. For example, one can attempt to indirectly change attitudes by focusing on their antecedents: beliefs (Ajzen 1991). This can take the form of linking the attitude object (here, the environment) with other objects or concepts (e.g., fragility, finiteness, etc.) or by providing support or evidence for such links (Fishbein and Ajzen 1972). Thus, information campaigns aimed at influencing individuals’ beliefs about the environment may be effective in promoting more positive environmental attitudes. In addition, one’s own experiences have been said to have an influence on attitudes. Fishbein and Ajzen (1972) note, for example, that, under some circumstances, interpersonal contact can also bring about belief and attitude changes. According to the Elaboration Likelihood Model, processes of attitude change also differ in terms of different routes of information processing (Petty and Cacioppo 1986).

A main limitation of our study is the fact that the behavior as well as willingness to sacrifice are based on self-reports. We do not have validation data and thus cannot establish whether their reported behavior is consistent with their actual behavior. However, as attitudes were shown to have no robust effects on reported consumption and mobility behavior, this is a moot point to some extent. In other words, had we found effects, it could have been argued that, say, social desirability led to respondents *saying *that their behavior had changed, although it had not. But because we are looking at whether individual-level changes in one’s attitudes relative to their usual attitudes influence changes in their behavior relative to their usual behavior, it does not make sense for a respondent driven by social desirability to report that their attitude toward the environment became more positive but their behavior stayed the same. Furthermore, because of the use of FE regression, if we can assume that the tendency toward social desirability is stable within individuals (Andersen and Mayerl 2019, p. 20), then it should be controlled for along with the other stable unobserved characteristics.

Willingness to sacrifice, in contrast with reported (past) behavior, is a prospective measure, and we should therefore expect less of a discrepancy between the reported willingness and the “true value,” i.e., what the respondent truly intends to do (the real gap we should expect between attitudes and behavior, not necessarily attitudes and intentions). On that point, it is again generally agreed that willingness to sacrifice represents a form of behavioral intention (Klösch et al. 2021; Knez et al. 2020; Luzar and Cossé 1998; Mayerl and Best 2019; Meyerhoff 2006). And if that were true, then it is worth mentioning that expressing an intention to behave in a given way generally does not guarantee that a person will indeed follow through. Importantly, however, in this case, we are talking about things that are generally *out of the hands of individuals*. A person can plausibly express the intention to drive his/her car less, and this is something that they have agency over. However, an individual cannot set his/her tax rate independent of their income, nor do they have any real say on the prices of, say, energy or groceries. And the items used here are clearly framed in terms of willingness to *accept* outside decisions, as opposed to willingness to make individual decisions contrary to narrow self-interests (Davis et al. 2009). In this way, the willingness to sacrifice measure describes a passive acceptance of decisions made by political actors. It tells us how an individual intends to act should the government decide to raise taxes to fight climate change, for example. It seems reasonable to believe that if someone states that they are willing to pay higher taxes if it is in the service of preserving the environment, that they will indeed accept a tax hike and not, contrary to their stated intention, join a political protest or switch political preferences in opposition to said taxes.

This study set out to take a careful look at the question of whether a change in attitudes can bring about a change in behavior. This goes above and beyond the question whether or not attitudes and behavior are correlated. Looking at the RE models, it is obvious that the two constructs do indeed correlate in our study. But our interest lies in the causal effect: if we could manipulate attitudes to become more positive in a given individual, holding all other characteristics stable, would we expect that person to begin to behave in a more environmentally friendly way? Indeed, throughout this article, we mentioned that our interest lies in answering this causal question. Fixed effects regression is an important tool available for looking at questions of causality, because it enables us to hold all unobserved time-invariant characteristics constant. This is a step in the right direction, but it does not guarantee that the observed effect can be given a causal interpretation. We attempted to reinforce our analysis by accounting for measurement error (which can lead to attenuation bias), and state dependence or habits. More work is needed to replicate our findings, perhaps also in an international setting, not just in the case of Germany. And it can never be guaranteed that some (time-varying) unobserved confounders, or interaction effects have been neglected that could negate the findings as they pertain to willingness to pay. However, we feel as though we have identified a relationship with potential policy relevance. And recent experience with the COVID-19 pandemic seems to support the idea that in crises, individuals cannot always be relied upon to make responsible decisions, at least anecdotally. Information campaigns aimed at creating more positive attitudes toward the environment, and the breaking down of anthropocentric views seem important to ensure the political continuity necessary to make large-scale contributions to climate change.

## Appendix

### Results of CFAs

**Table 6:** Confirmatory factor analysis, consumption behavior

Observed variable	Latent construct	Unstandardized factor loading	Standardized factor loading	SE	z‑value
Shpbiomrkt	Consumption behavior	1.000	0.650	–	–
Shprgnlprdct		0.920	0.598	0.013	71.309

Estimator: Diagonally weighted least squares, *n* = 1999, robust Chi-squared = 142.699, df = 25, *p* value (Chi-squared) = 0.000. Robust fit measures: Comparative Fit Index = 0.996, Tucker–Lewis Index = 0.994, Root Mean Squared Error of Approximation = 0.049, Standardized Root Mean Square Residual = 0.015

Metric and threshold longitudinal measurement invariance (factor loadings and thresholds). Standardized factor loadings based on the initial point in time as they vary slightly over time

*SE* standard error

**Table 7:** Confirmatory factor analysis, mobility behavior

Observed variable	Latent construct	Unstandardized factor loading	Standardized factor loading	SE	z‑value
Usgsbusrgn	Mobility behavior	1.000	0.816	–	–
Usgsbuslngrng		0.789	0.644	0.037	21.366
Usgscar		0.770	0.628	0.038	20.034
Usgsbike		0.379	0.309	0.032	11.819

Estimator: Diagonally weighted least squares, *n* = 1681, robust Chi-squared = 1765.126, df = 196, *p* value (Chi-squared) = 0.000. Robust fit measures: Comparative Fit Index = 0.985, Tucker–Lewis Index = 0.986, Root Mean Squared Error of Approximation = 0.069, Standardized Root Mean Square Residual = 0.076

Metric and threshold longitudinal measurement invariance (factor loadings and thresholds). Standardized factor loadings based on the initial point in time as they vary slightly over time

*SE* standard error

**Table 8:** Confirmatory factor analysis, willingness to sacrifice

Observed variable	Latent construct	Unstandardized factor loading	Standardized factor loading	SE	z‑value
Wtshrprcs	Willingness to sacrifice	1.000	0.877	–	–
Wtshrtxs		0.803	0.704	0.015	53.634
Wtslfstl		0.750	0.658	0.015	49.095

Estimator: Diagonally weighted least squares, *n* = 2164, robust Chi-squared = 246.947, df = 106, *p* value (chi-square) = 0.000. Robust fit measures: Comparative Fit Index = 0.997, Tucker–Lewis Index = 0.997, Root Mean Squared Error of Approximation = 0.025, Standardized Root Mean Square Residual = 0.013

Metric and threshold longitudinal measurement invariance (factor loadings and thresholds). Standardized factor loadings based on the initial point in time as they vary slightly over time

*SE* standard error

**Table 9:** Confirmatory factor analysis, environmental attitudes

Observed variable	Latent construct	Unstandardized factor loading	Standardized factor loading	SE	z‑value
Nepadptenvrnmt	Environmental attitudes	1.000	0.635	–	–
Nepblncntr		1.134	0.720	0.021	53.419
Nepenvcrsisexgrt		1.029	0.653	0.021	48.748
Nephmrlntr		1.026	0.651	0.021	49.242

Estimator: Diagonally weighted least squares, *n* = 2269, robust Chi-squared = 1391.345, df = 196, *p* value (Chi-squared) = 0.000. Robust fit measures: Comparative Fit Index = 0.972, Tucker–Lewis Index = 0.973, Root Mean Squared Error of Approximation = 0.052, Standardized Root Mean Square Residual = 0.036

Metric and threshold longitudinal measurement invariance (factor loadings and thresholds). Standardized factor loadings based on the initial point in time as they vary slightly over time

*SE* standard error

## Footnotes

1 Vicente et al. (2021) define a third type of behavior, “activist behavior”, which refers to individual participation in debates, involvement with formal organizations, etc. We do not focus on this type of behavior owing to the constraints posed by the available data.

2 Some notable exceptions known to us are Best and Kneip (2011, 2019) and Franzen and Vogl (2013).

3 Although it depends on what one is trying to explain, these and other preferences can also be classified as restrictions (Diekmann and Voss 2004, p. 16).

4 See Appendix: Results of CFAs.

5 See, for example, David Kenny’s overview on rules of thumb for evaluating model fit measures in SEMs: http://www.davidakenny.net/cm/fit.htm.

6 How plausible it is to consider such things as time-invariant heterogeneity depends mostly on the length of the observation period; over the course of a few days these almost certainly do not change for the vast majority of observed units; over the course of years or decades some of these are almost certainly time-varying. In our case, with an observation period of 5 years, a number of things could indeed change over time.

7 Note that even if *w* correlates with *z*, the explicit modelling of *w* as a latent variable will block this potential back-door path as well.

8 Waves bc, cc, dc, ec, fc, gc.

9 For more methodology information concerning the GESIS Panel, please see Bosnjak et al. (2017).

10 See https://www.gesis.org/gesis-panel/documentation, under “Wellenberichte,” report for wave gc.

11 The observed variables are ordered categorically, so below we use the diagonally weighted least squares estimator with robust standard errors and mean- and variance-adjusted test statistic in lavaan. Full information maximum likelihood (FIML) is widely regarded as one of the most robust ways to deal with missing data (Allison 2002), but it cannot be implemented for categorically observed variables in current SEM software. We ran all models again considering the observed variables as continuous with FIML and found no discrepancies in the results in terms of significance and sign of effects.

12 We tested another model that accounted for reverse causality, i.e., an effect of previous behavior on current attitudes, as suggested by Allison et al. (2017). However, the results did not differ from those of the DFE model with regard to sign and significance.

13 The standardized coefficients are not shown in the tables owing to space constraints.

14 They actually found robust within-unit effects of attitudes on behavior, but their analysis differed from ours in that they did not look at lagged relationships, nor did they account for habitual behavior or explicitly address measurement error in the variables of interest.

15 For an overview, see Fishbein and Ajzen (1972), Petty and Cacioppo (1986) and Petty and Wegener (1999).
